# Osteoporosis and the Potential of Cell-Based Therapeutic Strategies

**DOI:** 10.3390/ijms21051653

**Published:** 2020-02-28

**Authors:** Iratxe Macías, Natividad Alcorta-Sevillano, Clara I. Rodríguez, Arantza Infante

**Affiliations:** Stem Cells and Cell Therapy Laboratory, Biocruces Bizkaia Health Research Institute, Cruces University Hospital, Plaza de Cruces S/N, 48903 Barakaldo, Bizkaia, Spain; IRATXE.MACIASGARCIA@osakidetza.eus (I.M.); NATIVIDAD.ALCORTASEVILLANO@osakidetza.eus (N.A.-S.)

**Keywords:** MSCs, Osteoporosis, immunomodulation, cell-based therapy, bone turnover markers

## Abstract

Osteoporosis, the most common chronic metabolic bone disease, is characterized by low bone mass and increased bone fragility. Nowadays more than 200 million individuals are suffering from osteoporosis and still the number of affected people is dramatically increasing due to an aging population and longer life, representing a major public health problem. Current osteoporosis treatments are mainly designed to decrease bone resorption, presenting serious adverse effects that limit their safety for long-term use. Numerous studies with mesenchymal stem cells (MSCs) have helped to increase the knowledge regarding the mechanisms that underlie the progression of osteoporosis. Emerging clinical and molecular evidence suggests that inflammation exerts a significant influence on bone turnover, thereby on osteoporosis. In this regard, MSCs have proven to possess broad immunoregulatory capabilities, modulating both adaptive and innate immunity. Here, we will discuss the role that MSCs play in the etiopathology of osteoporosis and their potential use for the treatment of this disease.

## 1. Introduction

Osteoporosis, a skeletal disorder characterized by low bone mass and increased bone fragility, has no clinical manifestations until a fracture occurs. Osteoporosis is normally diagnosed in elderly patients, being increasingly recognized as a major public health concern [1]. In 2013 22 million women and 5.5 million men in Europe were affected, representing approximately 6% of men and 21% of women aged 50–84 years. These numbers are seriously escalating over time due to population aging [2].

Bone is an organ in constant remodeling, finely orchestrated by two principal cell types: osteoclasts that degrade the bone matrix (bone resorption) and osteoblasts which synthesize a new bone matrix (bone formation). In osteoporosis, there is an imbalance between bone resorption and bone formation [3] mainly due to three mechanisms responsible for developing fragile bone tissue: failure to achieve peak bone mass, excessive bone resorption and/or inadequate formation of new bone during remodeling [4]. The bone mass of an individual in adulthood depends on the peak bone mass reached during the growth period and the subsequent rate of bone loss [5]. The achieved maximum bone density in adults is crucial since it contributes to bone density to a larger extent than bone loss rate until at least 15 years after menopause [6]. Therefore, failure to reach a peak of high bone mass in the first three decades of life would contribute significantly to development of osteoporosis [7]. A healthy skeleton requires constant remodeling of bone tissue. Under normal conditions, there is a balance between bone formation and resorption, which is necessary to maintain the bone mass and its mechanical resistance properties. This balance is achieved by closely regulating the activity of osteoblasts and osteoclasts, responsible for bone formation and resorption, respectively. However, under certain pathological conditions an imbalance between bone resorption and bone formation may occur, leading to abnormal bone remodeling and the development of bone disorders like osteoporosis.

The diagnosis of osteoporosis is performed by image analysis (principally X-ray and densitometry) but nowadays, there is increasing interest in the use of bone remodeling biochemical markers for clinical applications in bone metabolic disorders. These bone turnover markers (BTM) are substances released into the circulation during the bone formation and/or resorption process, which reflect the metabolic activity of the tissue at a specific time and, therefore, their potential in the evaluation of bone metabolic disorders. The ideal bone remodeling marker should meet the following characteristics: non-invasive determination, ease of sample collection, be an specific product of bone metabolism, show correlation with reference techniques in the bone remodeling analysis such as bone histomorphometry, bone biopsy after double labeled with tetracycline, isotopic studies with labeled calcium and/or dual x-ray absorptiometry and respond to the specific treatment of diseases that affect bone metabolism [8].

The identification and analysis of these markers is vital for the monitoring of osteoporosis as well as for other bone metabolic diseases, since the use of bone turnover markers allows following the progression of the disease and in addition the therapeutic response of the patient. Moreover, these markers have the capacity to predict the risk of fracture and therefore can be useful in the adoption of certain therapeutic decisions. Thus, several prospective studies examining the relationship between BTMs and subsequent fractures showed that one or more markers of bone turnover (formation or resorption) were significantly associated with fracture risk [9,10,11]. Furthermore, in women with a low bone mineral density, the presence of an increase of certain BTMs has an additive effect on fracture risk [12,13,14,15].

## 2. Bone Turnover Markers (BTM)

A summary of the main bone turnover markers that exist, their peculiarities and their potential in osteoporosis management are detailed below.

### 2.1. Markers of Bone Resorption

#### 2.1.1. Amino and Carboxyl-Terminal Cross-Linking Telopeptides of Type I Collagen (NTX and CTX)

Type I collagen is the most abundant protein component of bone, representing 90% of the organic extracellular matrix [16]. Type I collagen is formed by a triple helix of two molecules of pro-α1 chain, codified by *COL1A1* gene; and one pro-α2 chain, encoded by *COL1A2*. During bone resorption process, collagen is degraded into different fragments. C- and N-terminal telopeptides of type I collagen (CTX and NTX, respectively) are both fragments from the telopeptide region, a non-triple-helical portion near the ends of mature collagen molecule. Telopeptides are cleaved during osteoclastic resorption of bone and, are released into the blood stream at a rate which is proportional to bone resorption activity [17].

Two types of proteinases have been described to take part in this process; the cysteine proteinases, which act at acidic pH and matrix metalloproteinases (MMP) that act at neutral pH. Thus, depending on the acting proteinase, one telopeptide molecule or another is released. CTX and NTX are generated from the activity of the cysteine proteinase cathepsin K, while the MMP or trypsin digestion of bone, leads to the release of cross-linked telopeptide of type I collagen (ICTP) [18]. Actually, CTX, NTX and ICTP molecular markers of type I collagen degradation have been shown to respond differently according to the clinical situations and treatments. This is due to the difference in the enzymatic pathways leading to their release. ICTP levels have been reported to respond more to pathways of bone resorption activated by skeletal metastasis of malignant tumors, multiple myeloma and rheumatoid arthritis [17,19] whereas CTX has been proposed by International Osteoporosis Foundation (IOF) to be used as a reference marker for bone resorption, in the context of fracture risk and therapy monitoring in osteoporosis [20]. There are diverse assays for measuring CTX, both in urine and in serum, including enzyme-linked immunosorbent assay (ELISA), radioimmunoassay (RIA) and an electrochemiluminescence assay [21]. Importantly, CTX levels show a circadian variation with a maximum at 05:00 h and a minimum of about 14:00 h [22]. This circadian variation is only affected by fasting, which significantly reduced this variation, thus the collection of the sample is recommended in the morning after overnight fasting [23].

NTX can also be measured in serum or urine, although it is preferentially measured in urine; since urine NTX is more sensitive than serum NTX in detecting changes induced by antiresorptive therapies [24]. To avoid the variability due to circadian changes in bone turnover, NTX is measured in 24-hour urine samples by ELISA immunoassays (using antibodies that recognize the α2 crosslinked fragment of type I collagen). Besides, NTX levels are less sensitive to dietary intake changes compared to CTX.

#### 2.1.2. Pyridinoline (PYD) and Deoxypyridinoline (DPD) Cross-Links

Pyridinoline (PYD) and deoxypyridinoline (DPD) are covalent pyridinium cross-links that bridge several collagen peptides and mechanically stabilize the collagen molecule [25]. They are produced from the breakdown of collagen during bone resorption and their levels strictly reflect the degradation of mature crosslinked collagens. PYD and DPD are released into circulation and subsequently excreted in urine either as free or peptide-bound moieties.

PYD is found in numerous tissues such as cartilage, bone, ligaments and vessels, while DPD is only detected in bone and dentin. In any case, the turnover of the bone is much higher than in the aforementioned tissues, so it is considered that the PYD and DPD of both, serum and urine, are produced mostly in the bone tissue. Moreover, since PYD and DPD levels are not altered by food intake, pyridinium crosslinks are viewed as good markers of bone resorption. Both free and conjugated forms of PYD and DPD have been shown to be stable in urine samples kept at room temperature for several weeks. If storage occurs at −20 °C they can last for years, the repeated freeze-thaw cycles of urine samples have no effect on their concentrations [26].

Pyridinium cross-links can be detected and quantified by automated high-performance liquid chromatography (HPLC) [27], direct immunoassays for free and peptide-bound forms [28,29], as well as by liquid chromatography tandem mass spectrometry (LC–MS/ MS) [30].

#### 2.1.3. Hydroxyproline (OHP)

Hydroxyproline (OHP), an amino acid formed from the post-translational hydroxylation of proline, constitutes 12–14% of the total amino acid content of mature collagen [31]. During the bone degradation process, OHP is released and 90% of it reaches the liver, where it is metabolized and finally excreted in the urine, either in free form or linked to peptides [32]. Hydroxyproline can be measured by colorimetric assays or HPLC methods [33,34,35].

Although urinary OHP is considered a bone resorption marker it should be taken cautiously since significant amounts of OHP in the urine proceed from newly synthesized procollagen during bone formation process [36]. Furthermore, hydroxyproline can be found in other tissues like skin and cartilage, being also released from the metabolism of elastin and complement component 1q (C1q) protein [37,38]. These drawbacks prevent OHP from being considered as a specific marker for bone resorption and it has been replaced by other biomarkers that show higher specificity in osteoporosis monitoring [39].

#### 2.1.4. Hydroxylysine-Glycosides

Hydroxylysine is produced during the synthesis of collagen due to the post-translational modifications suffered by collagen. Subsequently, hydroxylysine can undergo additional modifications like glycosylation giving rise to two different forms; glucosyl-galactosyl-hydroxylysine (GGHL) and galactosyl-hydroxylysine (GHL). The glycosilation of the hydroxylysine is different depending of the tissue; GHL is specific for bone, while GGHL is the major form in skin [40]. Although hydroxylysine is found in all collagens, GHL is five-to seven fold more concentrated in type I collagen of bone than in type I collagen of skin [41].

Both of them are released into the circulation during collagen degradation and can be measured in the urine by HPLC after derivatization with a fluorescence compound [42]. Later, an immunoassay for urinary GHL was developed based on polyclonal antibodies [43].

The glycosylated forms of hydroxylysine are not metabolized before excretion in urine, as the case with hydroxyproline, nor are they affected by dietary intake. It has been estimated that urinary hydroxylysine glycosides represent the 50–100% of collagen breakdown, a much higher percentage than the estimated for OHP [44]. Therefore, since the 90s it has been regarded as a better marker than OHP [45,46].

#### 2.1.5. Tartrate-Resistant Acid Phosphatase (TRACP5b or TRAP5b)

This enzyme belongs to the ubiquitous family of the acid phosphatases. There are five isoforms which are expressed in different cell types. All acid phosphatases are inhibited by L (+)-tartrate, except form 5, so the latter it was called tartrate resistant acid phosphatase (TRAP or TRACP). There are two TRACP subforms—type 5a, which contains sialic acid and type 5b, which does not, the latter being characteristic of osteoclasts [47]. In fact, this TRACP5b is accepted as a marker of both: the number of osteoclasts and their activity [48,49]. It presents low diurnal variability and is not affected by feeding [50], being typically increased in high bone turnover conditions [51]. There are specific immunoassays for serum TRACP5b detection, actually it has been proposed for the evaluation of BMD in women [52].

TRACP5b stability goes as follow: serum samples can be kept for 2 days at room temperature and for 3 days at 4 °C. However, for longer storage, freshly collected samples should be stored in aliquots at −20 °C for a maximum of one month or at −70 °C to remain stable for years [53].

#### 2.1.6. Bone Sialoprotein (BSP)

Bone sialoprotein, an important extracellular glycoprotein of mineralized tissues, represents the 5–10% of the non-collagenous matrix of bone. Although its theoreticalmolecular weight (MW) is 33 kDa, due to different post-translational modifications (N and O-linked glycosylation, serine and threonine phosphorylation, tyrosine sulfation and sialylation) it has an apparent MW of 60–80 kDa [54]. BSP is produced by many different cell types during bone morphogenesis such us osteoblasts, osteoclasts, osteocytes and hypertrophic chondrocytes [55,56]. In fact, this protein is considered to play an important role in cell-matrix adhesion processes and in the supramolecular organization of the extracellular matrix of mineralized tissues [41].

Serum BSP can be measured by different immunoassays [57]. Several studies show that BSP levels might be a useful marker in laboratory assessment of bone turnover since it shows a significant correlation to established markers of bone formation like bone alkaline phosphatase (B-ALP) and osteocalcin (OC) [58], to bone resorption markers such as PYD, DPD, NTX as well as bone resorptive cytokines interleukin-11 (IL-11) and transforming growth factor β2 (TGFβ2) [59]. Based on the rapid reduction of serum BSP levels following intravenous bisphosphonate treatment, it is considered to reflect bone resorption processes [31].

#### 2.1.7. Cathepsin K

Cathepsin K is a protein of 329 amino acids form by a long N-terminal signal sequence of 15 amino acids, a 99 amino acid long propeptide and a 215 amino acid long catalytic unit [60]. This protein is a member of the cysteine protease family, being able to cleave both helical and telopeptide regions of type I collagen [61]. Cathepsin K is predominantly expressed in osteoclasts and other multinucleated cells. Immunocytochemical studies have shown that it is located in various intracellular compartments of the osteoclasts such as vesicles, granules and vacuoles. Later, it is secreted into the extracellular environment, specifically into the bone resorption lacuna underneath actively resorbing osteoclasts, where it is responsible for the degradation of type I collagen [62]. Therefore, cathepsin K and specifically its circulating form, may be a useful and specific biochemical marker of osteoclastic activity [31]. Likewise, it has been demonstrated its relevance for osteoporosis, given that bone resorption associated with cysteine protease and cathepsin K by osteoclasts is negatively regulated by estrogens [63]. Moreover, the osteoclastogenic factor receptor activator for nuclear factor kappa B ligand (RANKL) appears to directly up-regulate cathepsin K expression [64]. There are several immunoassays available to measure cathepsin K levels in serum samples [65]. In addition, it is also possible to quantify the activity of the protein by means of a synthetic substrate that, when metabolized, releases a fluorescent compound [66].

### 2.2. Markers of Bone Formation

#### 2.2.1. Serum Osteocalcin

OC, also known as bone γ-carboxyglutamic acid-containing protein (BGLAP), is a non-collagen protein of 5.8 kDa. Although non-collagen proteins represent only 10% of the organic extracellular matrix, their roles in the regulation of bone turnover and mineralization are essential [67,68]. OC is synthesized by mature osteoblasts, being the most abundant non-collagenous bone matrix protein (15% of total non-collagen proteins) [69]. Although the function of OC is not completely elucidated, it might have a role in the regulation of osteoblasts function, as suggested by knockout mouse models. Surprisingly, at six months of age, OC^−/−^ mice showed a marked increase in bone formation, increased cortical and trabecular thickness, seeming their bones mechanically more stable than those of the wild type mice [70].

OC is often used as a marker for late bone formation [71]. It is synthesized and secreted by osteoblasts, later being incorporated in bone extracellular matrix. However, during this process a small fraction is released into the blood stream being detectable by immunoassay. Serum OC have shown to correlate with bone formation rate [72,73]. However, serum OC is rapidly degraded even at temperatures as low as 4 ·C, resulting in OC fragments that can be detected by antibody based assays along with the full-length molecule. The detection of intact OC or its fragments is an important issue to be aware of, since tests that detect only intact OC will be particularly sensitive to in vitro degradation, while tests that detect fragments may, according to the recognized fragments, overestimate the concentration of intact OC [74].

Serum OC presents a high biological and circadian variability, which might negatively affect the reproducibility of repeated measures. Either RIA, ELISA or a chemiluminescence immunoassay may be used to detect OC in serum [75,76].

#### 2.2.2. Serum Alkaline Phosphatase and Bone-Specific Alkaline Phosphatase

Alkaline phosphatases (ALPs) are plasma membrane-bound glycoproteins. Human ALPs can be classified in four tissue-specific isoforms; placental alkaline phosphatase (PLALP or Regan isozyme), intestinal alkaline phosphatase (IALP), tissue nonspecific alkaline phosphatase (TNAP) and germ cell ALP (GCALP or NAGAO isozyme) [77]. The tissue non-specific ALP gene encodes kidney, liver and bone isoforms of ALP. In healthy individuals, bone and liver specific ALP constitute 95% of the total ALP activity found in serum. In adults, both isoforms are normally present at equal proportion (1:1) [40]; however, during childhood and adolescence, due to skeletal growth, bone isoform is predominant (up to 90%) [78]. Bone specific ALP is generated by osteoblasts during bone formation. Although its exact function is not completely elucidated, it is known that ALP presence in osteoblasts cell membrane is necessary for bone mineralization [79] and ALP expression correlates positively with bone formation rate [80].

In order to differentiate between the two main circulating ALP isoforms, several techniques have been developed; namely, heat denaturation, electrophoresis, precipitation, selective inhibition and immunoassays [81]. Nonetheless, immunoassays show certain degree of cross-reactivity and subjects with high liver ALP, can result in artificially high bone ALP measurements, leading to false positive results [82].

#### 2.2.3. Serum PINP and PICP

N-terminal and C-terminal propeptide of type I procollagen (PINP and PICP, respectively), are released from posttranslational modifications that type I procollagen molecule suffers during collagen assembly into fibrils [83]. Both molecules are produced in equimolar amounts with each other and with the collagen deposited in the tissue.

PINP has 70 kDa and is rich in proline and hydroxyproline, which are essential for the triple helical structure stability. PINP is eliminated from the circulation by liver endothelial cells through their scavenger receptors [84]. PINP primarily originates from bone but other tissues like skin, tendon, dentin, ligaments, cartilage and interstitial tissue also contribute to PINP serum levels [85]. Serum PINP concentrations reflect the new skeletal bone formation, therefore, diseases that involve high bone turnover would be expected to be associated with high serum concentrations of PINP. Normally, PINP is present in serum in two forms; an intact trimeric form and a monomeric form. The two PINP forms have different catabolic routes; trimeric PINP is rapidly cleared from blood circulation by scavenger receptors on liver endothelial cells [84], while monomeric form clearance mechanism is still poorly understood. However, it is probably cleared via kidneys, as shown by the similar monomeric fragment of amino-terminal propeptide of type III collagen [86]. Both forms can be detected together by the “total” PINP assay whereas the “Intact” PINP assay, only detects the trimeric form [83]. In addition, the measurement can be performed by both automatic and manual methods like RIA, ELISA and electro-chemiluminescence immunoassays. Serum PINP is quite insensitive to food intake and the circadian rhythm, in clinical trials, this marker has been shown to increase dynamically in response to treatment with teriparatide [87], an anabolic drug that increases bone formation. PINP is stable in serum/plasma for at least five days at room temperature and for four weeks at 4 °C [88].

The other molecule—PICP—consists of 115 kDa and it is produced in the same way that PINP during collagen synthesis in bone formation processes. PICP is stabilized by disulphide bonds and is cleared by liver endothelial cells via the mannose receptor which can be regulated by growth and thyroid hormones [89]. PICP presents a short serum half-life of only 6–8 minutes, which limits its use as biomarker.

PINP, on the other hand, presents a longer stability in serum besides being no affected by food intake and circadian rhythm. All of that, point this molecule as a remarkable biomarker. In fact, PINP has been recommended as the bone formation marker by the International Osteoporosis Foundation (IOF) and the International Federation of Clinical Chemistry (IFCC) for clinical research studies in osteoporosis [90]. Likewise, the National Bone Health Alliance has also recommended PINP as the reference marker for bone formation [91].

## 3. Osteoporosis Treatments

A healthy and active lifestyle is vital for the proper maintenance of all body tissues, including bone. Several studies have highlighted the importance of physical exercise to improve the quality of life of patients with osteoporosis [92,93,94]. Recently, Filipović, T. et al. 2020 [95], has demonstrated that exercising is able to modulate the enzymatic activity of serum matrix metalloproteinase-9 (MMP-9) and tissue inhibitor of metalloproteinase 1 (TIMP-1) in postmenopausal osteoporotic patients.

Diet also plays a fundamental role in bone health. Calcium supplementation has been shown to be able to decrease the rate of bone mineral density loss in women, although it does not seem to be enough to prevent fractures [96]. The combination of calcium with vitamin D showed better results, with a reduction of 15% in the risk of total fractures and 30% in the risk of hip fractures [97]. During menopausal transition, the drop of endogenous estrogens enhances bone resorption, at the expenses of the new bone formation, leading to osteoporosis [98]. In consequence, estrogen intake could be an appropriate therapy against osteoporosis. However, estrogen therapy is associated with elevated cancer risk in estrogen receptor (ER)-α rich tissues like the endometrium, breast and ovary. Therefore, alternative molecules have been sought to prevent this unwanted effect of estrogen therapy. In that matter, isoflavones are a type of phytoestrogens that have shown a weak binding to ERα [99] and preferentially bind to ER-β; present in bone, liver, heart and brain. Therefore, they mimic the effects of estrogens in some tissues and at the same time, block the estrogen effects in others. Isoflavones can be absorbed through the diet in legumes (most importantly in soy), nuts and some fruits. Recently, in a randomized clinical trial, isoflavone treatment has achieved a decline in the BMD loss, together with an increased in bone turnover. Moreover, the combination of this phytoestrogens with calcium, magnesium and calcitriol showed even better results [100]. However, its effectiveness remains controversial since a meta-analysis of 10 long term clinical trials concluded that soy isoflavones did not show significant improvement in lumbar spine, total hip or femoral neck BMD of postmenopausal women [101]. Altogether, these approaches are insufficient to prevent the progression of osteoporosis. Therefore, pharmacological therapies have been developed to counteract bone fragility based on molecular targets [102]. Therapies for osteoporosis are focus on restoring the normal balance between bone resorption and bone formation. Currently, the most common therapies are the anti-resorptive ones, focusing on the inhibition or reduction of bone resorption process. These agents include estrogens, selective estrogen receptor modulators (SERMs), bisphosphonates and monoclonal antibodies [103].

Bone resorption is highly determined by hormone levels, such us estrogens. As mentioned above, unopposed estrogen can increase the risk of breast and uterine cancers, deep vein thrombosis and stroke [104]. Therefore, in order to reduce the side effects of estrogens, SERMs have taken a step forward. Although they demonstrate selectivity toward estrogen receptors in the bone and are able to maintain bone mineral density (BMD), they lack the efficacy of traditional estrogens [105]. Many clinical trials with postmenopausal osteoporotic women using different types of SERMs have shown that their benefit for fractures prevention is anatomically limited (present certain limitations in preventing non-vertebral fractures). In addition, SERMs are also associated with detrimental extra-skeletal effects such as an increased risk of a cardiovascular event and endometrial cancer risk [106], as well as related side effects such as thromboembolic events and, in some cases, carcinogenesis [107].

Among anti-resorptive drugs, the most used are bisphosphonates. Bisphosphonates are pyrophosphate analogues that bind to hard bone through their affinity for hydroxyapatite [108]. They are incorporated into bone matrix and taken up by osteoclasts, suppressing their activity in bone remodeling [109]. This way, the bone density increases but the quality of the bone is compromise since the old bone is prone to have microfractures that negatively affect its function. Moreover, the prolonged use of bisphosphonates may lead to adverse events, such as gastrointestinal problems, osteonecrosis of the jaw, atrial fibrillation and musculoskeletal pain [110,111].

Denosumab is a monoclonal antibody that binds with high affinity to receptor activator of nuclear factor kappa-Β ligand (RANKL), preventing the binding between RANKL and receptor activator of nuclear factor kappa-Β (RANK) and thus inhibiting the differentiation and activity of the osteoclasts [112]. Unlike bisphosphonates, Denosumab is not incorporated into bone, yielding a much shorter terminal half-life [113]. Consequently, Denosumab presents a potential advantage for the patients who have a side effect for the therapy, due to the fact that it will be no longer active six months after the last dose. A big concern regarding Denosumab treatment is the regular adhesion to this treatment, because the patient’s fracture risk might increase after the dose “wears off” [114].

Although anti-resorptive osteoporosis medications reduce fractures, they have rare and serious adverse effects that may limit their safety for medium and long-term use, so new safe therapies capable of restoring skeletal structure and integrity are needed. In fact, current pharmacologic attempts for osteoporosis aim to prevent fractures through stimulation of bone formation. These agents target anabolic pathways to stimulate the osteoblastic activity, increasing the bone volume without inhibiting its resorption.

The first approved agent to accomplish this was teriparatide. Teriparatide is a recombinant human parathyroid hormone (PTH), known to be the only available therapeutic agent that increases the formation of new bone tissue [115]. PTH regulates the amount of calcium in bone; therefore this treatment is used to stimulate osteoblasts to create new bone [116,117]. Toxicological studies revealed osteosarcoma in treated rats; nonetheless this problem has not been detected in treated patients. As a consequence, the approved lifetime duration of treatment with teriparatide is 24 months; but it is recommended only for patients for whom potential benefits outweigh potential risk [118,119].

Romosozumab, is another monoclonal antibody recently approved by the U.S. Food and Drug Administration (FDA) for the treatment of osteoporosis in postmenopausal women. The registered trade name of Romosozumab is Evenity. It is the first humanized anti-sclerostin monoclonal antibody that has been shown to increase bone formation with dual effect: on one hand, it increases bone formation and, on the other, although to a lesser extent, it reduces bone resorption (or bone loss) which translates into a decrease in the risk of fracture [120]. However, according to the prescribing information; it may increase the risk of myocardial infarction, stroke and cardiovascular death. Therefore, it should not be administered to patients who have had a myocardial infarction or stroke within the preceding year.

Sclerostin, expressed by osteocytes and articular chondrocytes, is the product of SOST gene. It is an endogenous inhibitor of the Wingless-type mouse mammary virus integration site (Wnt) signaling pathway. Wnt signaling has been described as a positive regulator of bone formation and regeneration [121] and thus, Wnt signaling could be modulated to treat osteoporosis and other skeletal diseases associated with low BMD and increased facture risk [122]. Therefore, anti-sclerotin compound would inhibit sclerostin (an inhibitor of Wnt signaling) and in consequence, promote Wnt signaling and stimulate bone formation by osteoblasts [123]. Another two anti-sclerostin monoclonal antibodies are being developed by other companies; blosozumab (Eli Lilly and Company, Indianapolis, IN, USA) and BPS804 (Novartis, Basel, Switzerland).

In summary, currently used osteoporosis therapies are not fully effective in all patients and present considerable side effects that seriously compromise their long-term use. Thus, the development of new therapeutic strategies for osteoporosis is craved in an increasingly aging world population with a longer life expectancy.

## 4. Mesenchymal Stem Cells

As mentioned before, current attempts for developing safe new therapies are focused on anabolic therapeutic strategies, able to increase the activity of new bone forming cells (osteoblasts) and/or their progenitor.

The osteoblasts progenitors, named as mesenchymal stem cells (MSCs) are spindle shaped cells with multipotent differentiation capacity in vitro. The International Society for Cellular Therapy (ISCT) has proposed minimum criteria to define MSCs. These cells (i) should exhibit ex vivo plastic adherence under normal conditions (ii) possess specific immunophenotype, such as the expression of CD73,CD90,CD105 and lack of CD14, CD34, CD45 and human leukocyte antigen-DR (HLA-DR) (iii) have the ability to differentiate into osteogenic, adipogenic and chondrogenic lineages in vitro [124].

The study of MSCs and the pathways involved in adipogenesis, osteogenesis and chondrogenesis [125], have contributed to the understanding of the mechanism that leads into an osteoporotic phenotype.

In healthy individuals, the rate of MSCs in bone marrow is very low (about 0.01 %–0.001 %) [126], being even lower in aged population. If, in addition to this, the aged MSCs lose their capacity to suitably proliferate and differentiate being the subsequent bone formation compromised, there is a high risk of developing osteoporosis [127]. Moreover, osteoporosis could be caused, at least in part, due to the preferential differentiation of MSCs into adipocytes instead of osteoblasts in the aging skeleton [128]. Many different studies have compared the functional characteristics of MSCs from osteoporotic patients versus controls; MSCs from osteoporotic women present a lower growth rate than control cells [129], together with decreased TGF-β production and decreased capacity to generate and maintain a type I collagen-rich extracellular matrix [130]. Both conditions support the hypothesis that MSCs from osteoporotic patients have an increased tendency to differentiate into adipocytes [131]. Supporting this observation, cells derived from osteoporotic donors have diminished ALP activity [129] and calcium deposition under osteogenic differentiation conditions. 

MSCs fate determination is regulated by specific transcription factors [132,133] and intrinsic signals [125], such as runt-related transcription factor 2 (Runx2) [134] and osterix (Osx) for osteoblasts differentiation; and peroxisome proliferator activated receptor gamma (PPARγ) for adipocytes [135,136]. Interestingly, these regulators have been found to be altered in osteoporotic MSCs leading to functional alterations that result in poor osteogenic differentiation capacity and increased adipogenesis [137,138].

In addition, hMSCs from patients with osteoporosis and age-matched controls have been shown to exhibit different transcriptomes. Osteoporotic MSCs show an enhanced mRNA expression of genes involved in osteoclastogenesis (VEGF, TGFB and CSF1) and genes coding for inhibitors of Wnt and bone morphogenetic protein (BMP) signaling, indicating intrinsic deficiencies in self-renewal and differentiation potential of osteoporotic stem cells [139].

## 5. Therapeutic Potential of Mesenchymal Stem Cells

Given the diminished proliferation and differentiation ability of MSCs from osteoporotic patients, it is quite reasonable to contemplate the benefits of providing healthy MSCs to the patients to boost the bone anabolic pathway. Numerous efforts have been attempted in the field of cellular therapy based on MSCs applied to osteoporosis. In the beginnings, the cell therapy potential was focus on MSCs´ ability to target a damaged site and subsequently, to differentiate into osteoblasts in order to repair the degenerated tissue. However, the poor engraftment demonstrated by transplanted cells in humans [140] generates a serious questioning regarding the limitation of this mechanism of action. A more consensus mechanism of action relays on their ability to act in a paracrine manner by secreting factors that modify the environment and recruit resident cells to repair the degenerated tissue [141].

However, a recent murine study [142] has demonstrated the capability of healthy Bone-Marrow derived MSCs to engraft in osteogenesis imperfecta (OI) mouse model improving bone phenotype. OI is a rare bone disorder characterized by bone fragility like osteoporosis. The direct intra-bone transplantation of bone-marrow MSCs achieved long-term engraftment (up to 6 months post-transplantation), with the consequent improvement of cortical structure and strength in mouse bone. The greater efficiency of this study compared to the one mentioned above [140] may be due to the mode in which MSCs are administered considering that, in the previous treated OI pediatric patients MSCs were intravenously infused. Thus, different routes of MSCs administration should be further investigated in order to improve MSCs effectiveness in the treatment of bone metabolic disorders.

### 5.1. Mesenchymal Stem Cells from Different Origins as a Novel Therapy for Osteoporosis

The MSCs used to treat osteoporosis could have different origins: Bone-Marrow derived MSCs (BM-MSCs), adipose-tissue derived MSCs (AD-MSC) or umbilical cord derived MSCs (UC-MSCs). Although all of them have shown therapeutic effects for osteoporosis in preclinical studies, there are still some challenges on their clinical application, such as the limited long-term survival and the uncertainty of MSCs´ fate after transplantation depending on the source of MSCs [140,143].

#### 5.1.1. Bone Marrow Derived MSCs (BM-MSCs)

Several studies in animal models intended to prove the potential of transplanted BM-MSCs to treat osteoporosis, both from allogenic and autologous sources.

Different osteoporotic murine models have been used to demonstrate the therapeutic capacity of MSCs. Ichioka and collaborators [144] used an irradiated P6 substrain of senescence-accelerated mice (SAMP6), which is an osteoporotic mouse model that exhibits age-dependent restraint of osteoblastogenesis and osteoclastogenesis along with enhanced adipogenesis. The authors demonstrated that locally administration of normal murine allogeneic BM-MSCs could increase trabecular bone and attenuate BMD loss [144].

Ovariectomy (OVX)-induced rat model has also been used to challenge the potential of MSCs to treat osteoporosis. Allogenic BM-MSCs isolated from healthy rats were injected into the femurs of osteoporotic rats. The femurs of treated rats showed trabecular bone percentage almost similar to the femurs from control healthy rats. As expected, osteoporotic BM-MSCs showed less differentiation capabilities as compared to those isolated from healthy rats [145].

Glucocorticoid-induced osteoporosis mouse model has also been applied to study the effect of the systemic injection of allogeneic BM-MSCs. The encouraging outcomes comprised inhibition of the decrease of bone mass and strength as well as stimulation of osteoblastogenesis, with consequent sustained bone formation [146].

Finally, Kiernan, Hu, Grynpas, Davies, & Stanford (2016) [147] made use of an age-related osteoporosis mouse model, Sca-1^−/−^, to demonstrate that systemic administration of allogeneic bone marrow MSCs, noticeably improved bone formation and maintained the microarchitectural capability.

In addition, further non-murine animal models have been used to study the beneficial effect of BM-MSCs injection to treat osteoporosis. Autologous BM-MSCs had been transplanted in an OVX-induced rabbit model of osteoporosis, displaying more bone apposition, stronger stiffness of bone, raised trabecular thickness and developed microstructures with freshly formed osteoids [127]. Similar effects were obtained when BM-MSCs were injected in goats with long-term estrogen deficiency (mimicking the postmenopausal osteoporosis that occurs in humans) [148].

Phase I clinical trial is currently ongoing, where autologous fucosylated (a type of glycosylation) BM-MSCs are infused into osteoporotic patients. However, there is some uncertainty regarding the use of autologous BM-MSCs for osteoporosis treatment in elderly patients, due to the age-related decline in the overall BM-MSC number [143,149] (ClinicalTrials.gov Identifier: NCT02566655).

#### 5.1.2. Adipose Tissue-Derived MSCs (AD-MSCs)

Adipose tissue-derived MSCs have become increasingly popular in many stem cell applications since they are more easily isolated, more abundant and produce higher yields in terms of cell number compared with BM-MSCs [150]. However, the efficiency of AD-MSCs and their proliferative and differentiation capacities vary depending on the tissue harvesting site [151] and in a similar way to BM-MSCs, on the donor age [152].

Different animal models had also been utilized to study the efficiency of AD-MSCs to treat osteoporosis. SAMP6 age-related osteoporotic mice had been transplanted with isogenic AD-MSCs, resulting in a significant improvement in several trabecular bone parameters [153]. Cho and collaborators focused on preventing OVX-induced bone loss in nude mice by injection of human AD-MSCs; reassuring remarkable rise in BMD was seen in the mice [154].

Additional encouraging studies based on OVX-induced rabbit models of osteoporosis transplanted with autologous AD-MSCs shown enhanced bone regeneration, due to their capacity to stimulate osteogenesis and prevent adipogenesis of osteoporotic BM-MSCs. These encouraging outcomes were achieved through activation of bone morphogenetic protein 2 (BMP-2) and the bone morphogenetic protein receptor type 1B (BMPR-IB) signal pathway [155].

#### 5.1.3. Umbilical Cord Derived MSCs (UC-MSCS)

BM- and AD-MSCs are effective sources of MSCs but their therapeutic potential can be affected by the donor’s lifestyle and age. Thus, bone regenerative medicine has focused its efforts on finding new sources of MSCs in perinatal tissues, such us umbilical cord [156]. These cells are younger than adult BM-and AD-MSCs, have strong osteogenic differentiation ability, demonstrated few immunogenic adverse effects and have an easy and noninvasive harvesting procedure without any risk to the donor [157,158].

Bone formation by umbilical cord-derived MSCs (UC-MSCs) was demonstrated in vivo by Diao and collaborators who loaded human UC-MSCs into scaffolds that were transplanted subcutaneously into BALB/c nude mice, observing efficiently bone formation by UC-MSCs [159]. An et al. (2013) [160] used OVX nude mice as a model to study the influence of systemic injection of human umbilical cord blood hUCB-MSCs. After the infusion, the BMD levels enhanced considerably and the micro-CT analysis of the mice tibiae exhibited considerably greater values of trabecular number and thickness, as well as bone volume.

So far, there are no clinical trials with UC-MSCs cells applied to osteoporotic patients.

## 6. Bone Turnover and Inflammation

Emerging clinical and molecular evidence suggests that inflammation exerts significant influence on bone turnover, thereby in osteoporosis. Estrogen deficiency is the main cause of osteoporosis in post-menopausal women. This condition results in increased production of inflammatory cytokines, describing osteoporosis as an inflammatory disease [161,162,163]. Estrogen has a protective effect in bone, due to a direct action on osteoclasts and osteoblasts. Regarding osteoclasts, estrogens significantly increase their apoptosis [164,165] and reduce the RANKL-dependent osteoclast formation [166]. In osteoblasts, they exert an anabolic effect by at least increasing osteoblast survival and collagen type I production [167]. Estrogens has been demonstrated to also modulate lymphocytes; its presence suppresses RANKL production in T and B lymphocytes [168], the lack of estrogens increases the release of pro-osteoclastogenic cytokines (tumor necrosis factor α (TNF-α) and RANKL) by activated T lymphocytes [169,170] and are also associated to an increase in B lymphocytes number [171].

Bone fractures repair is mediated not only by the bone but also with the aid of the immune system, being crucial in the process [172]. In fact, human immunodeficiency virus (HIV) patients, who are immunologically compromised, show a delay of fracture healing process [173] and B and T cell depletion has been reported to be associated with bone regeneration impairment, due to a reduction in osteoblast differentiation and bone mineralization [174].

The first phase of bone healing is characterized by an acute inflammatory response; the release of interleukin-1 (IL-1), interleukin-6 (IL-6) and TNF-α recruits B and T lymphocytes. T lymphocytes have a pro-osteogenic role, by releasing interleukin-17F (IL-17F) [175]. This cytokine promotes Col1a1, osteocalcin and sialoproteins in osteoblasts [175]. After that, immune cells produce chemoattractant molecules like CXCL7 (NAP2) and monocyte inflammatory protein (MIP)-1 alpha. Once bone repair process has started, the inflammatory response must be stopped to avoid more damage. To this end, MSCs stimulate the differentiation of the T-reg lymphocytes, inducing the apoptosis of the pro-inflammatory Th1 and Th17 lymphocytes and inhibiting migration of B cells.

## 7. MSCs Immunoregulatory Capabilities

MSCs have been shown to possess immunoregulatory abilities [176], being capable of interacting with cells of both adaptive and innate response: B cells, T cells, dendritic cells (DCs), natural killer (NK) cells, neutrophils and macrophages [177]. These immunomodulation relies on cell-cell contact in collaboration with the secretion of soluble immune factors [178] namely; TGFβ1, hepatocyte growth factor (HGF), indoleamine-2, 3-dioxygenase, prostaglandin E2 (PGE2), interleukin-10 (IL-10), HLA-G5 and galectins.

MSCs have been broadly demonstrated to be able to modulate the differentiation, function and balance of T cells subpopulations (Th1, Th2, Th17 or Tregs) and promote the development of an anti-inflammatory immune response [179]. Indeed, MSCs constitutively secrete B7-H4 molecule and human leukocyte antigen G (HLA-G), which presents an immunosuppressive action on T lymphocytes, hindering proliferation and cellular mediated cytotoxicity [180]. With respect to adaptive humoral immunity, MSCs reduce B-cell proliferation by inducing cell cycle arrest in the G0/G1 phase [181]. They can also affect B-cell differentiation and immunoglobulin synthesis (IgM, IgG and IgA) by the release of metalloproteinase-processed CC-chemokine ligand 2 (CCL2) [182,183].

MSCs have been demonstrated to exert an immunosuppressive function on dendritic cells (DCs), which are considered the bridge between innate and adaptive immune system. They can inhibit DCs differentiation and maturation in a reversible manner [184,185]. MSCs have also the ability to decrease the production of the pro-inflammatory cytokines (such as interleukin-12 (IL-12) [186] and TNF-α), as well as to up-regulate the production of the anti-inflammatory cytokine IL-10 in monocytes [187]. Therefore, MSCs are able to impair both the antigen presentation function and the pro-inflammatory potential of DCs. In fact, allogeneic UC MSCs seems to suppress inflammation in lupus erythematosus patients though up-regulating tolerogenic DCs [188].

As for the innate immune system, some studies have shown that MSCs are capable of inhibiting NK cell proliferation, cytokine release and cytotoxicity, via prostaglandin E2 and indoleamine dioxygenase [187,189]. The inhibitory effect on the effector functions of NK cells is produced by a sharp down-regulation of the surface expression of the activating NK receptors NKp30, NKp44 and NKG2D. Later, Thomas and co-workers 2014 [190] showed that in the proper conditions, MSCs may also support NK cell function. MSCs enhance the IFN-γ secretion by NK cells in the presence of the pro-inflammatory cytokines IL-12 and IL-18. Both cytokines are frequently expressed during the immune response to pathogens. Therefore, it is possible that MSCs, when present at the site of infection support the elimination of invading pathogens through the stimulation of NK cells for increased IFN-γ production. But not only NK cells, MSCs also support neutrophils function by promoting their viability and enhancing their recruitment and activity in proinflammatory situations. MSCs, even at very low proportions in vitro (MSC: neutrophils at 1: 500), are able to inhibit apoptosis of both, resting neutrophils and IL-8 activated ones. In addition, they reduce the respiratory burst induced by N-formyl-l-methionine-l-leucyl-l-phenylalanine (f-MLP) without affecting other processes like phagocytosis, expression of adhesion molecules or neutrophil migration capacity in response to classical stimuli [191]. In addition, this study also showed that MSCs by constitutive release of IL-6 rescued neutrophils from apoptosis. Another study in mice showed that when tissue-resident MSCs recognize microbial molecules it results in increased production of growth factors, such as IL-6, interleukin-8 (IL-8), granulocyte-macrophage colony-stimulating factor (GM-CSF) and macrophage migration inhibitory factor (MIF), that recruit neutrophils and enhance their pro-inflammatory activity [192]. In a later study, in a murine model of sepsis it was shown that the infusion of MSCs helped bacterial elimination through the improvement of phagocytic activity of neutrophils [193].

Lastly, increasing evidences have shown that the regulation of macrophages by MSCs is essential for the inflammatory response and tissue lesions repair. It has been reported that MSCs can interact with monocytes and macrophages to reprogram them [194]. When MSCs are activated by pro-inflammatory signals (produced by M1 macrophages or activated T cells), they can introduce two negative feedback loops into the generic pathway of inflammation [195]. In one loop, the activated MSCs secrete prostaglandin E2 (PGE2) that drives resident macrophages with an M1 pro-inflammatory phenotype towards an M2 anti-inflammatory phenotype [196]. It has been demonstrated that when macrophages and MSCs are cocultured, the production of M2 macrophages is induced. Hence, the phagocytic activity and secretion of IL-10 is upregulated and levels of inflammatory cytokines (interferon γ (IFNγ), TNF-α, IL-1β and IL-12) downregulated [197]. This way, MSCs modulate the preferential shift of the macrophage phenotype from M1 to M2 [198]. In the second loop, there is a negative feedback that let MSCs to serve as regulators of the early phases of inflammation. When MSCs are activated, they secrete TNFα stimulated gene/protein 6 (TSG-6) that interacts with macrophages to decrease TLR2/NFκ-B signaling, so a decrease in the secretion of pro-inflammatory mediators occurs [199]. These data underline the importance of the interactions between MSCs and the innate immune system in balancing pro-inflammatory and anti-inflammatory responses in order to preserve tissue integrity [200].

In summary, these results demonstrate the regulatory role of MSCs in the innate and adaptive immunity, due to their ability to down-regulate the intensity of an immune response. Since inflammation seems to significantly contribute to the etiopathogenesis of osteoporosis [201]; it is plausible to think, based on previous data that MSCs could help to decrease inflammation in osteoporosis patients.

## 8. Final Considerations

Osteoporosis is a skeletal disorder increasingly recognized as a major public health problem. Current osteoporosis treatments are no safe for long-term use, so the development of new therapeutic strategies is needed. MSCs are increasingly considered as promising tools as a new therapeutic strategy to treat osteoporosis, mainly due to its ability to secrete factors that are directly or indirectly involved in bone repair; as well as, its ability to graft into tissues and differentiate into functional osteoblasts. Emerging evidence suggests that inflammation exerts significant influence on bone turnover, thereby in osteoporosis. In fact, MSCs also have the capacity to interact with different immune cells, so the immunomodulatory capacity of MSCs could be an interesting approach to treat osteoporosis.

## Abbreviations

AD-MSC	Adipose-tissue derived MSCs
ALPs	Alkaline phosphatases
B-ALP	Bone alkaline phosphatase
BGLAP	γ-carboxyglutamic acid-containing protein
BMD	Bone mineral density
BM-MSCs	Bone-Marrow derived MSCs
BMP	Bone morphogenetic protein
BMP-2	Bone morphogenetic protein 2
BMPR-IB	Bone morphogenetic protein receptor type 1B
BSP	Bone sialoprotein
BTM	Bone Turnover Markers
CCL2	Metalloproteinase-processed CC-chemokine ligand 2
CTX	Carboxil-terminal cross-linking telopeptides of type I collagen
DCs	Dendritic cells
DPD	Deoxypyridinoline
ELISA	Enzyme-linked immunosorbent assay
FDA	Food and Drug Administration
f-MLP	formyl-l-methionin-l-leucyl-l-phenylalanine
GCALP	Germ cell alkaline phosphatase
GGHL	Glucosyl-galactosyl-hydroxylysine
GHL	Galactosyl-hydroxylysine
GM-CSF	Granulocyte-macrophage colony-stimulating factor
HGF	Hepatocyte growth factor
HIV	Human immunodeficiency virus
HLA-DR	Human leucocyte antigen-DR
HLA-G	Human leukocyte antigen G
HPLC	High-performance liquid chromatography
IALP	Intestinal alkaline phosphatase
ICTP	Cross-linked telopeptide of type I collagen
IFCC	International Federation of Clinical Chemistry
IFN*γ*	Interferon*γ*
IL-1	Interleukin-1
Il-10	Interleukin-10
IL-11	Interleukin-11
IL-12	Interleukin-12
IL-17F	Interleukin-17F
IL-6	Interleukin-6
IL-8	Interleukin-8
IOF	International Osteoporosis Foundation
ISCT	International Society for Cellular Therapy
LC–MS/ MS	Liquid chromatography tandem mass spectrometry
MIF	Macrophage migration inhibitory factor
MIP	Matrix metalloproteinases
MMP	Monocyte Inflammatory Protein
MSCs	Mesenchymal Stem Cells
NK	Natural killer
NTX	Ntx-Amino-terminal cross-linking telopeptides of type I collagen
OC	Osteocalcin
OHP	Hydroxyproline
OI	Osteogenesis imperfecta
Osx	Osterix
OVX	Ovariectomy
PGE2	Prostaglandin E2
PICP	C-terminal propeptide of type I procollagen
PINP	Pinp-N-terminal propeptide of type I procollagen
PLALP	Placental alkaline phosphatase
PPARγ	Peroxisome proliferator activated receptor gamma
PTH	Human parathyroid hormone
PYD	Pyridinoline
RANK	Receptor activator of nuclear factor ҝ Β
RANKL	Receptor activator for nuclear factor ҝ B ligand
RIA	Radioimmunoassay
Runx2	Runt-related transcription factor 2
SAMP6	Samp6-P6 substrain of senescence-accelerated mice
SERMs	Selective estrogen receptor modulators
TGF-β	Transforming growth factor β
TGFβ2	Transforming growth factor β2
TNAP	Tissue nonspecific alkaline phosphatase
TNF-α	Tumor necrosis factor α
TRACP5b or TRAP5b	Tartrate resistant acid phosphatase
TRAP or TRACP	Tartrate resistant acid phosphatase type 5b
TSG-6	TNFα stimulated gene/protein 6
UC-MSCs	Umbilical cord derived MSCs
Wnt	Wingless-type mouse mammary virus integration site
